# Novel Insights into the Genetic Controls of Primitive and Definitive Hematopoiesis from Zebrafish Models

**DOI:** 10.1155/2012/830703

**Published:** 2012-07-25

**Authors:** Raman Sood, Paul Liu

**Affiliations:** Oncogenesis and Development Section, National Human Genome Research Institute, National Institutes of Health, Bethesda, MD 20892, USA

## Abstract

Hematopoiesis is a dynamic process where initiation and maintenance of hematopoietic stem cells, as well as their differentiation into erythroid, myeloid and lymphoid lineages, are tightly regulated by a network of transcription factors. Understanding the genetic controls of hematopoiesis is crucial as perturbations in hematopoiesis lead to diseases such as anemia, thrombocytopenia, or cancers, including leukemias and lymphomas. Animal models, particularly conventional and conditional knockout mice, have played major roles in our understanding of the genetic controls of hematopoiesis. However, knockout mice for most of the hematopoietic transcription factors are embryonic lethal, thus precluding the analysis of their roles during the transition from embryonic to adult hematopoiesis. Zebrafish are an ideal model organism to determine the function of a gene during embryonic-to-adult transition of hematopoiesis since bloodless zebrafish embryos can develop normally into early larval stage by obtaining oxygen through diffusion. In this review, we discuss the current status of the ontogeny and regulation of hematopoiesis in zebrafish. By providing specific examples of zebrafish morphants and mutants, we have highlighted the contributions of the zebrafish model to our overall understanding of the roles of transcription factors in regulation of primitive and definitive hematopoiesis.

## 1. Zebrafish as a Model for Hematopoiesis

Recently, zebrafish have emerged as a powerful vertebrate model system due to their external fertilization, optically clear embryos, rapid development, availability of tools for manipulations of gene expression during development, and the ability to generate genetic mutants by random (insertional and chemical) and targeted mutagenesis [1–3]. Microinjections of antisense morpholinos, which cause transient knockdown of gene activity, and mRNA allows for analysis of the effects of loss and gain of function of specific genes during development [4]. Whole-mount in situ hybridization (WISH) is a powerful technique to analyze the spatiotemporal expression of genes, and placing genes in regulatory cascades by analysis of genetic mutants and/or embryos injected with morpholinos (commonly termed as morphants) [5, 6].

Specifically for hematopoiesis, zebrafish blood contains cells of all hematopoietic lineages [7–11] and orthologs of most transcription factors involved in mammalian hematopoiesis have been identified indicating evolutionarily conserved pathways of regulation [12–15]. Initial validation of the use of zebrafish for hematopoiesis research came from the forward genetic screens. In 1996, two large-scale chemical mutagenesis screens were performed to identify mutants with a variety of phenotypes [16, 17]. Of these, characterization of 46 mutants with blood phenotypes by allelic complementation suggested roles for at least 26 genes in hematopoiesis [18, 19]. Subsequent efforts by several groups identified the underlying genetic defects in many of these mutants by positional cloning or candidate gene approaches. In addition to identifying the genes previously known to have a role in hematopoiesis (e.g., *gata1*, *sptb*, and, *alas2*), these mutants also uncovered novel genes with roles in hematopoiesis, (e.g., *slc25a37*, *slc40a1,* and *glrx5*) [20–25]. Subsequent forward genetic screens focusing on mutants affecting specific hematopoietic lineages have identified additional conserved pathways of regulation between zebrafish and mammals [26–28]. 

This led to a surge of activity in zebrafish research laboratories, developing a variety of tools for thorough analysis of hematopoiesis. Lineage-specific transgenic lines were generated using promoters of a variety of hematopoietic genes driving fluorescent markers (reviewed in [29, 30] and listed in Table 1), allowing for visual observations of hematopoietic lineages in real-time during development. Advances in imaging combined with the ability to perform lineage tracing made it possible to follow the fate of specifically marked cells during development in a live vertebrate animal model [31, 32]. Sorting of hematopoietic cells by fluorescence-activated cell sorting (FACS), *in vitro* culturing using zebrafish-specific cytokines and kidney stromal cells, and the ability to perform transplantation have facilitated characterization of hematopoietic potential of different mutants [33–37].

While forward screens are biased by the phenotype being screened, mutants in any specific gene can be generated using reverse genetic approaches. This has been made possible in zebrafish in the last decade by TILLING (Targeting-Induced Local Lesions IN  Genomes) [55, 60], and more recently by targeted mutagenesis using zinc-finger and transcription-activator-like-effector nucleases (i.e. ZFNs and TALENs) [61–64]. Furthermore, effects of gene dosage can be analyzed by injecting suboptimal doses of antisense morpholinos or studying hypomorphic alleles generated by TILLING. In this review, we discuss how the technical advances and genomic tools discussed above went hand-in-hand with the elucidation of genetic controls of hematopoiesis in zebrafish.

## 2. Ontogeny of Vertebrate Hematopoiesis

In mammals, hematopoiesis occurs in successive but overlapping waves that occur at distinct anatomical locations [65]. Overall, the hematopoietic process is distinguished into primitive and definitive hematopoiesis based on the type of blood cells generated. Primitive hematopoiesis is transient in nature and produces unipotent blood cells that arise directly from the mesoderm. Definitive hematopoiesis produces multipotent blood cells that give rise to multiple different lineages through cellular intermediates and support blood cell development throughout the life of the organism. Here, we have summarized the overall process of mammalian hematopoiesis based on the studies using mouse models. 

During embryogenesis, primitive hematopoiesis occurs in two distinct waves in the extraembryonic yolk sac blood islands, producing primitive macrophages and primitive erythrocytes, respectively, thus providing the developing embryos with oxygen and their first line of defense against pathogens [66]. There is some support for the presence of additional lineages, particularly megakaryocytes, during primitive hematopoiesis [67].

Definitive hematopoiesis also occurs in two distinct waves. The first wave of definitive hematopoiesis produces a transient population of cells, termed erythroid-myeloid progenitors (EMPs) in the yolk sac and fetal liver [68, 69]. The second wave of definitive hematopoiesis produces hematopoietic stem cells (HSCs) from the hemogenic endothelium of the embryo that includes the aorta-gonad-mesonephros (AGM) region of the embryo, yolk sac, and placenta [65, 70–72]. HSCs from these sites migrate through circulation to fetal liver to support hematopoiesis during embryogenesis [65, 70, 73]. Recently, Chen and colleagues [74] demonstrated that EMPs and HSCs are derived from two different hemogenic endothelial populations. Unlike HSCs, EMPs lack the potential to give rise to lymphocytes.

The site of adult hematopoiesis, where HSCs undergo differentiation to generate lineage-committed progenitors that give rise to all the mature blood cell types and self-renewal to maintain a constant supply of HSCs, is bone marrow [75]. The prevailing thinking, based on the current data, is that HSCs emerging from the hemogenic endothelial cells in the AGM region of the developing mouse embryo give rise to most (if not all) bone marrow hematopoietic cells [73, 76]. The shifting sites of hematopoiesis are thought to provide specific microenvironment cues required for the specification, and migration of precursors for lineage commitment [77, 78]. 

Although the overall process of hematopoiesis is well defined, we have just begun to elucidate the exact nature of the molecular controls and lineage relationships using *in vitro* colony assays and animal models, particularly mice and zebrafish. The key questions revolved around the generation, migration, and differentiation of HSCs into lineage-committed progenitors and how these processes are regulated to maintain a critical balance required for proper functioning of the hematopoietic system.

### 2.1. Primitive Hematopoiesis in Zebrafish

In zebrafish, the first blood cells can be observed in circulation at around 26 hours post fertilization (hpf). However, based on the expression patterns of the genes involved in primitive hematopoiesis, it is clear that the primitive hematopoiesis starts at ~11 hpf in the lateral plate mesoderm (LPM) during somitogenesis. The erythroid precursors are observed as bilateral stripes in the posterior lateral mesoderm (PLM) that fuse along the midline to form the intermediate cell mass (ICM) located in the trunk dorsal to the yolk tube extension by 24 hpf [29, 75, 77, 79–81]. Primitive myeloid progenitors initiate at the anterior lateral mesoderm (ALM) and differentiate into macrophages in the rostral blood island [80, 82]. Thus, primitive hematopoiesis in zebrafish occurs in two waves, producing primitive macrophages and primitive erythrocytes, respectively. In addition, neutrophils and thrombocytes have also been detected during primitive hematopoiesis in zebrafish. However, the origin of neutrophils during primitive hematopoiesis is not clear, as two recent reports presented contradictory data on their origin from either primitive macrophage lineage [83] or primitive erythrocyte lineage [84] using fate-mapping techniques. Thus, primitive blood cells in zebrafish appear to have diverse lineages, similar to the mouse [67]. However, further studies are required to clearly define the lineage relationships between these cell types during primitive hematopoiesis. 

### 2.2. Definitive Hematopoiesis in Zebrafish

The hallmark of definitive hematopoiesis is generation of multipotential HSCs that can undergo self-renewal and differentiation to produce cells of erythroid, myeloid, and lymphoid lineages. In zebrafish HSCs can be identified by their expression of *runx1* and *cmyb* as early as 26 hpf in the ventral wall of the dorsal aorta and hence this region of the embryo is referred to as the AGM [13, 29]. Two recent studies have unequivocally demonstrated the origin of HSCs from the hemogenic endothelium lining the ventral wall of the dorsal aorta using time lapse imaging and lineage tracing in double transgenic lines marking HSCs and endothelial cells with different fluorescent markers [47, 85]. A novel process of cell transition, termed endothelial hematopoietic transition (EHT), appeared to be involved in the production of HSCs from hemogenic endothelium [85]. Similar to the mouse, a transient multipotent progenitor population of EMPs supports definitive hematopoiesis during embryogenesis and these EMPs originate in the posterior blood island (PBI) of zebrafish [86].

The sites of adult hematopoiesis in zebrafish are kidney marrow (analogous to the mammalian bone marrow) and thymus (for T cells) [13, 29, 87]. Up until recently, a site analogous to mammalian fetal liver was not recognized in the zebrafish. Therefore, HSCs from AGM were presumed to support embryonic definitive hematopoiesis and migrate to thymus and kidney for adult definitive hematopoiesis. However, two independent studies demonstrated the existence of an intermediate site of hematopoiesis posterior to the yolk tube extension, termed caudal hematopoietic tissue (CHT), using imaging and cell tracing techniques [88, 89]. It was proposed that the function of CHT is analogous to that of the fetal liver in mammals for supporting definitive hematopoiesis during embryogenesis. By tracing the generation and migration of HSCs using cd41:GFP^low^ cells, Kissa and colleagues [90] validated the migratory route of HSCs as being AGM to CHT and then to thymus and pronephros. Recently, Hess and Boehm [91] elegantly imaged the process of thymopoiesis in real time in zebrafish using triple transgenic lines and their data suggested that AGM is a major source of thymus-settling lymphoid progenitors compared to CHT.

Thus, based on the current status of our understanding, definitive hematopoiesis in zebrafish occurs in two waves: first wave produces transient EMPs in the PBI region and second wave produces HSCs in the AGM region that migrate to CHT to support larval definitive hematopoiesis and to thymus and kidney marrow to support adult definitive hematopoiesis. It is not clear if the migration of HSCs from AGM to kidney and thymus is via CHT only or also occurs directly as was previously assumed.

## 3. Elucidation of Genetic Controls of Hematopoiesis in Zebrafish

Despite the spatial and temporal differences during hematopoiesis between zebrafish and mammals as discussed above, the overall process is highly conserved producing the same effective repertoire of hematopoietic cells. It begins from a cell, termed hemangioblast, that serves as a common precursor for hematopoiesis and vasculogenesis [92, 93]. A complex network of regulatory signals is involved in the specification and lineage commitment of precursors during primitive and definitive hematopoiesis in mammals. These include homeobox, notch, vegf, and wnt signaling pathways as well as specific transcription factors, such as Tal1 (Scl), Lmo2, Gata1, Cmyb, Runx1, Spi1 (Pu.1), and Ikzf1 (Ikaros), which are shown to function in a hierarchical manner [5, 94–99]. The importance of proper functioning of these transcription factors is evident from the preponderance of mutations and genomic rearrangements disrupting their activity detected in several blood disorders, particularly leukemias and lymphomas [100–106].

Animal models, where level of gene activity can be manipulated, have played a critical role in advancing our understanding of the genetic controls of hematopoiesis. However, knockout mice are embryonic lethal at mid-to-late gestation for *Tal1*, *Lmo2*, *Gata1*, *Sfpi1 (Pu.1)*,* Myb*, and *Runx1*, thus precluding the examination of their roles in later stages of hematopoiesis [107–112]. Conditional knockout is a useful tool to determine the function of these genes later in life; however, it has been difficult to use this technology to study the initiating events of a lineage, especially for the HSCs, since appropriate promoters to drive Cre recombinase expression may not be available. Zebrafish provide an advantage over mouse models due to their ability to survive without blood for several days and are, therefore, a suitable model organism for investigating the effects of loss of function of genes that cause embryonic lethality in mice due to the hematopoietic defects. Here, we discuss the contributions of zebrafish mutants, morphants, and transgenic lines to our understanding of the regulatory cascade controlling the hematopoiesis process (Table 1 lists the lineage-specific transgenic lines and genetic mutants in transcription factors involved in regulation of hematopoiesis). The common theme in the studies reviewed below is utilization of the unique features of zebrafish embryos and available tools for analysis of the disruptions to the gene activity in an effort to understand the overall process. 

### 3.1. Genes Involved at the Hemangioblast Level: *tal1* and *lmo2*

Based on their expression in both hematopoietic and endothelial cells, and the phenotypes of loss of function animal models, the T-cell acute lymphocytic leukemia 1 (*TAL1*) and the LIM domain only 2 (*LMO2*) genes are both believed to function at the hemangioblast level [12, 113]. Both genes were identified from translocations occurring in T-cell acute lymphoblastic leukemia, *TAL1* from translocation t(1;14) and *LMO2* from translocation t(11;14) [102, 104]. TAL1 is a basic helix-loop-helix (bHLH) transcription factor where the bHLH domain is involved in DNA binding as part of a multiprotein complex that includes LMO2 as a bridging protein. LMO2 belongs to the LMO family of zinc-finger proteins that are characterized by 2 LIM domains, each composed of 2 zinc fingers [104]. Knockout mice for *Tal1 *and *Lmo2* died *in utero* by embryonic days 9.5–10.5 (E9.5-10.5) due to lack of embryonic erythropoiesis [108, 112]. Thus, their roles during definitive and adult hematopoiesis were investigated by *in vitro* colony assays, chimeric mice, and/or conditional knockout mice [114, 115]. Failure to produce any myeloid colonies *in vitro* from *Tal*1^−/−^ yolk sac cells indicated a block at the EMP level [108]. Using conditional knockout mice, Hall and colleagues [114, 116] demonstrated that adult hematopoiesis can occur independent of Tal1 function with minor defects in erythropoiesis and megakaryopoiesis. On the other hand, Lmo2 was shown to be absolutely necessary for adult hematopoiesis based on the analysis of chimeric mice derived from *Lmo*2^−/−^ embryonic stem cells [115]. 

In zebrafish, *tal1 *is expressed in the ALM and PLM from ~11 hpf and in the posterior ICM at 26 hpf, validating its role in primitive hematopoiesis [39, 117, 118]. First direct proof for the exact site of HSC initiation between the dorsal aorta and the posterior cardinal vein being analogous to AGM in zebrafish came from the examination of Tg(tal1-PAC-GFP) embryos by time lapse imaging [40]. Loss-of-function analyses for *tal1* have been performed using morpholinos and a genetic truncation mutation, K183X, which deletes the bHLH domain [38, 119–121]. Homozygous mutant embryos (*tal*1^*K*183*X*/*K*183*X*^) exhibited lack of expression of markers of both primitive and definitive lineages and also lacked visible circulation at 26 hpf [38]. These studies not only confirmed the role of Tal1 during primitive hematopoiesis, but also provided direct evidence for the role of Tal1 in the initiation of definitive hematopoiesis. However, mutant embryos died due to pericardial edema and defects in heart morphogenesis and could not be studied for the role of Tal1 in transition of embryonic to adult stages of definitive hematopoiesis. 

In zebrafish,* lmo2* expression in the ALM and PLM is detected about 20 minutes after the *tal1* expression and phenotype of *lmo2* morphants is very similar to the *tal1* morphants, supporting their roles as part of the multiprotein complex during hemangioblast development [41, 122]. To date, no genetic mutants have been reported for *lmo2*. Overall, zebrafish studies have confirmed the strict requirements for Tal1 and Lmo2 in initiation of both primitive and definitive hematopoiesis. 

### 3.2. Genes Involved at the HSC Level: *runx1* and *cmyb*

The onset of definitive hematopoiesis in the AGM is marked by the specification of HSCs, which support hematopoiesis throughout the life of a vertebrate. *runx1* and *cmyb* have been used interchangeably as the earliest markers of definitive hematopoiesis due to their expression in the AGM during HSCs specification [12, 123]. However, we have just begun to elucidate their precise roles in HSCs specification, migration to the sites of larval and adult hematopoiesis, and differentiation into erythroid, myeloid, and lymphoid lineages. 

*RUNX1* belongs to a family of genes (3 members in mammals and 4 in zebrafish) that encode for the alpha subunits of a heterodimeric complex that binds DNA through the highly conserved runt domain. A single gene, *CBFB*, encodes for the beta subunit, which does not bind to DNA by itself but increases the affinity of alpha subunits to bind to DNA after heterodimerization through their runt domains [124]. Promoters of many hematopoietic genes, for example, *SPI1* and *GATA1*, contain RUNX1 DNA binding sites [125–127]. *RUNX1* was first identified in the t(8;21) translocation frequently observed in acute myeloid leukemias and its dimerization partner, *CBFB,* is also frequently involved in genomic rearrangements associated with leukemia [100, 128, 129]. Furthermore, mutations affecting the level of RUNX1 activity leading to loss of function, dominant negative gain of function, and/or overexpression are associated with other blood disorders such as familial platelet disorder with predisposition to acute myeloid leukemia and myelodysplastic syndrome, suggesting that the process of hematopoiesis is very sensitive to the level of RUNX1 activity [130–132]. 

Studies using knockout mouse models demonstrated that Runx1 is essential for the initiation of HSCs generation during definitive hematopoiesis as the mutant mice failed to develop fetal liver hematopoiesis and died *in utero* at E12.5 [111]. Conditional knockout mice were able to develop all lineages but showed defects in megakaryocyte maturation and differentiation of B and T cells [133, 134]. Recent elegant fate mapping experiments in mouse embryos by Chen and colleagues demonstrated that Runx1 is required for the emergence of HSCs from the hemogenic endothelium [135]. Taken together, these data suggest a strict requirement of Runx1 in the generation of HSCs to initiate definitive hematopoiesis and in further differentiation of certain lineages but not for the maintenance of HSCs if they are already produced (reviewed in [73]). 

Zebrafish *runx1* was identified based on its high similarity to the human *RUNX1* in the runt homology domain [123, 136]. Since then, several studies have validated the critical requirement of Runx1 in the initiation of definitive hematopoiesis by morpholinos and characterization of a variety of hematopoietic mutants [95, 97, 136, 137]. As these studies were performed prior to the recognition of CHT being the site of embryonic definitive hematopoiesis, they did not address Runx1 requirements in specification of EMPs and their transient nature precluded analysis of Runx1 requirements in adult hematopoiesis. None of the hematopoietic mutants from forward genetic screens mapped to the *runx1* locus. 

Therefore, our group performed TILLING to identify a truncation mutation, W84X, in the runt domain of *runx1* [42, 43]. Homozygous mutant embryos displayed a complete lack of cells expressing markers of HSCs, definitive erythroid, myeloid, and lymphoid lineages in the CHT and thymus between 3–5 dpf [42, 43]. However, utilizing Tg(cd41:GFP) transgenic zebrafish, we were able to demonstrate that cd41^+^ cells were formed in the *runx*1^*W*84*X*/*W*84*X*^ fish in the AGM and CHT regions and migrate to the pronephros, even though they were negative for other HSC markers such as *cmyb*. Based on the analysis of circulating blood cells, the mutant fish displayed 3 distinct phases: first phase of normal circulating blood cells until around 6–8 dpf (presumably from normal primitive hematopoiesis), second phase of bloodless stage until around 20 dpf leading to death in most larvae (defective larval definitive hematopoiesis), and astonishingly, ~20% of the mutant larvae resumed blood circulation and grew as phenotypically normal adult fish with multilineage adult hematopoiesis [43]. We do not know exactly how these 20% *runx1* mutant larvae were rescued. One possibility is that the cd41^+^ cells observed in these embryos are hematopoiesis-committed or -primed mesoderm cells, which could restart hematopoiesis in permissive conditions, such as compensation by *runx2a, runx2b,* and *runx3* genes or other genetic and/or epigenetic changes. Another scenario is that two waves of definitive hematopoiesis exist, one for larval and the other adult, while Runx1 is only required for the larval stage. For both scenarios, most larvae died due to lack of circulating blood cells resulting from defective larval hematopoiesis. It is interesting to note that alternate *runx1* promoters are used during establishment of EMPs and HSCs (Table 1) as demonstrated recently by Lam and colleagues [44].

Similarly, MYB, a cellular homolog of the V-MYB proto-oncogene, is a critical transcription factor required for definitive hematopoiesis. A number of mouse models, including conventional and conditional knockouts as well as hypomorphic alleles, have been generated for functional analysis of Myb requirements during hematopoiesis, as discussed in a recent review by Greig and colleagues [109]. These studies have highlighted the key difference between Runx1 and Myb requirements during definitive hematopoiesis to be the generation of HSCs. *Myb* knockout mice displayed defects in erythroid and myeloid development and died *in utero* at E15.5, which is much later than the stage when HSCs are generated [109]. Furthermore, *Myb* ^−/−^ ES cells were able to produce T cell progenitors in *Rag*1^−/−^ chimeric mice [138]. Thus, Myb deficiency causes a block in HSCs differentiation and lineage commitment rather than HSCs specification. Lieu and Reddy [139] demonstrated important contributions of Myb to self-renewal and differentiation of HSCs during adult hematopoiesis.

Recently, two groups reported characterization of loss of function mutants for *cmyb* in zebrafish: (1) allele t25127 with a missense mutation, I181N, affecting DNA binding domain and (2) allele hkz3, a splice site mutation leading to truncation of the transactivation domain. These mutants were identified from forward genetic screens for defects in thymopoiesis and lack of lysozyme C (*lyz*) expression, respectively [45, 46]. Homozygous embryos for either mutation showed lack of definitive hematopoiesis but behaved differently with respect to survival. *cmyb* ^*I*181*N*/*I*181*N*^ mutant embryos displayed severe anemia and became bloodless by 20 dpf. Although the mutants survived for 2-3 months with stunted growth, there were no detectable hematopoietic cells by FACS or histology [45]. This is in contrast to our finding with *runx*1^*W*84*X*/*W*84*X*^ mutants, thus suggesting differential requirements for *runx1* and *cmyb* activities during larval and adult hematopoiesis. On the other hand, most of the cmyb^*hkz*3^ mutants (splice site mutation affecting the transactivation domain) died by 10 dpf. The authors did not explain the reason for this difference. We speculate that the husbandry differences between laboratories might be the reason for their differential survival in the absence of blood cells. Using time-lapse imaging of *cmyb* ^*hkz*3^ */Tg(cd41:GFP)* embryos and lineage tracing, Zhang and colleagues [46] demonstrated an important role for *cmyb* in the migration of HSCs from ventral wall of the dorsal aorta (VDA) to CHT, thereby proposing that migratory defects of HSCs maybe the cause of failure of definitive hematopoiesis in *cmyb* deficient embryos. Thus, zebrafish models of *cmyb *deficiency have provided novel insights into its role in the migration of HSCs from AGM to CHT during definitive hematopoiesis.

### 3.3. Genes Involved at the Level of Erythropoiesis, Myelopoiesis, and Lymphopoiesis: *gata1*, *spi1*, and *ikzf1*

Differentiation of HSCs during definitive hematopoiesis into lineage-committed progenitors, which further differentiate into mature blood cells, is mediated by lineage-specific transcription factors [77]. Unlike HSCs, these lineage-committed progenitors lack the potential for self-renewal and thus require a constant supply of HSCs for their production [87, 140]. The first series of lineage-committed multi-potent progenitors are termed common myeloid and common lymphoid progenitors (CMPs and CLPs). In mammals, CMPs further differentiate into megakaryocyte-erythroid progenitors (MEPs) that produce mature erythrocytes and platelets (erythropoiesis), and granulocyte/macrophage progenitors (GMPs) for the generation of mature myeloid cells (myelopoiesis). CLPs produce mature lymphoid lineage cells (lymphopoiesis). However, intermediate multilineage progenitors have not been identified in zebrafish yet, and all lineage relationships are speculative. Here, we have summarized the genetic controls of erythropoiesis, myelopoiesis, and lymphopoiesis in zebrafish.

Erythropoiesis involves differentiation of erythroid-myeloid progenitors into mature erythrocytes and thrombocytes. The master regulator of erythropoiesis is GATA1, a transcription factor belonging to the GATA family (6 members) that contains a conserved DNA binding domain consisting of two zinc fingers [140, 141]. Its consensus DNA binding site, WGATAR, is found in regulatory regions of most erythroid-specific genes [142]. Human mutations in *GATA1* are associated with anemia, thrombocytopenia and acute megakaryoblastic leukemia in Down Syndrome patients [143]. *Gata1* knockout mouse embryos die by E10.5 due to severe defects in erythropoiesis during primitive hematopoiesis, precluding assessment of its role in definitive hematopoiesis without generating conditional knockout mice [107, 144]. 

 The zebrafish *gata1* gene was identified by cross-hybridization with the zinc-finger region of Xenopus *Gata1* [145]. Its expression is consistent with the sites of erythropoiesis during primitive hematopoiesis starting at 5-somite stage [49]. Using positional cloning of one of the bloodless mutants, termed *vlad tepes* or *vlt* ^*m*651^, identified in the 1996 large-scale forward screens, our group identified a truncation mutation, R339X, distal to the C-terminal zinc-finger domain in Gata1 [23]. As expected, homozygous mutant embryos displayed defects in primitive erythropoiesis and lacked visible circulating blood cells at the onset of circulation. Evaluation of definitive hematopoiesis by WISH revealed similar defects in erythropoiesis but normal development of myeloid and lymphoid lineages, thus demonstrating the specific role of Gata1 in generation of erythroid progenitor cells not only during primitive but also during definitive hematopoiesis [23, 48]. 

Myelopoiesis involves differentiation of erythroid-myeloid progenitors into differentiated macrophages/monocytes, mast cells, and granulocytes, including neutrophils and eosinophils [9, 80, 82]. The master regulator of myelopoiesis is *SPI1 *(previously known as *PU.1)*, an oncogene originally identified as the site of genomic rearrangements by spleen focus-forming proviral insertion in erythroblastic tumors [103]. SPI1 belongs to the ETS family of transcription factors that bind DNA through a purine rich sequence, termed the PU box [146]. *Sfpi1* knockout mice died around E18 due to multilineage defects, implicating additional roles of *Sfpi1* in erythropoiesis and lymphopoiesis [110]. *In vitro* studies have demonstrated the importance of a negative cross-regulation of Gata1 and Sfpi1 during erythroid and myeloid differentiation from CMPs [140]. Unlike mammals, the sites of erythropoiesis (PLM) and myelopoiesis (ALM) are separate in zebrafish during embryogenesis [50, 51]. However, upregulation of myelopoiesis in *gata1* morphants and ectopic expression of *gata1* in *spi1* morphants proved that similar cross-regulation of these two transcription factors is critical for the proper commitments of erythroid and myeloid lineages in zebrafish [147, 148].

Lymphopoiesis involves differentiation of lymphoid progenitors into mature T and B cells that participate in a functional immune system of the organism [11]. Primary lymphoid organs for T-cell maturation in zebrafish are bilateral thymii, which are marked by expression of *rag1*, *ikzf1* and *lck* starting at ~72 hpf [56, 57, 59]. Pancreas has been suggested as an intermediate site for the production of B cells [149] between 4 dpf to ~3 weeks, at which point B cells become evident in the kidney. However, this remains to be verified, as no good transgenic markers of B cells currently exist to follow their development in real time. The master regulator of lymphopoiesis is the transcription factor IKZF1 (previously known as IKAROS) [150]. IKZF1 contains six zinc-fingers that are involved in DNA binding and protein-protein interactions [151]. By analysis of knockout mice, Wang and colleagues [152] demonstrated differential requirements of Ikzf1 for B- and T-cell differentiation during fetal and adult hematopoiesis. *Ikzf1* null mice displayed complete blockage of differentiation of B cells during both fetal and postnatal stages. On the other hand, they displayed blockage of differentiation of T cells only during the fetal stage. Postnatal T-cell development recovered, albeit with deregulation of CD4 versus CD8 lineage commitment. Overall, their data suggested that *Ikzf1* is essential for lymphopoiesis (both B and T cells) during fetal hematopoiesis, but it is dispensable for adult T cell development. Similar to the knockout mice, zebrafish with a truncation mutation, Q360X, in *ikzf1* (*ikzf*1^*t*24980^), which removes the C-terminal two zinc fingers essential for protein-protein interactions, are adult viable [58]. Mutant fish displayed complete lack of lymphopoiesis during larval stage, and partial recovery after 14 dpf. Although the mutant fish survived and lived up to at least 17 months in nonsterile conditions, they displayed abnormal and inefficient lymphoid development. However, it is interesting to note that similar to our observations of two phases of definitive hematopoiesis in *runx1* mutants, zebrafish lacking Ikzf1 activity potentially demonstrated two phases of lymphoid development. In both cases, the larval phase is gene activity dependent while the adult phase develops to some extent despite the lack of gene activity. 

## 4. Different Activity-Levels, Domains, and Isoforms of the Same Transcription Factors Are Required during Different Stages of Hematopoiesis

Recent studies have demonstrated the need to address dosage requirements of transcription factors in the hematopoietic cascade as opposed to a simple on versus off situation [153–156]. In zebrafish, it is relatively easy to manipulate gene dosage by careful tuning of morpholino doses and generation of hypomorphic alleles using TILLING. Therefore, differential requirements for some of the transcription factors either in terms of level of activity or different isoforms have been demonstrated recently in zebrafish, as discussed below.

### 4.1. *Tal1*

As discussed previously, Tal1 plays critical roles during both primitive and definitive hematopoiesis. Using different doses of morpholinos to completely or partially abolish Tal1 activity, Juarez and colleagues [120] demonstrated differential requirements of *tal1* expression for erythroid specification and maturation during primitive hematopoiesis. Their work showed that lower activity of Tal1 was sufficient for primitive erythroid specification but not their maturation. Furthermore, by complementation experiments with wild-type and DNA-binding mutant forms of Tal1, they demonstrated differential requirements for the DNA-binding activity of Tal1 during erythroid specification and maturation. Their data suggested different mechanisms of target gene regulation during erythrocyte specification and maturation by Tal1: direct binding to promoters of the target genes involved in erythroid maturation and indirect regulation through other protein complexes for genes involved in erythroid specification. 

 Further complexity to Tal1 requirements during primitive and definitive hematopoiesis became obvious from the analysis of its two isoforms: the full-length form termed Tal1-*α* and a shorter form lacking the first 146 amino acids, termed Tal1-*β*. Using morpholinos to specifically target the *α* and *β* forms, Qian and colleagues [157] demonstrated that both forms act redundantly in initiation of primitive hematopoiesis, while only the Tal1-*β* form is required for the specification of HSCs in the AGM to initiate definitive hematopoiesis. Ren and colleagues [158] examined the requirements of Tal1-*α* and Tal1-*β* during angioblast and HSC specification, also demonstrating the requirement for Tal1-*β* in HSC specification. Thus, zebrafish research has contributed significantly to our understanding of the regulation of different stages of hematopoiesis by Tal1.

### 4.2. *Gata1*

Similar to Tal1, Gata1 activity is crucial for erythropoiesis during both primitive and definitive hematopoiesis. Recently, we described a hypomorphic allele of Gata1 due to a missense mutation, T301K, in its C-terminal zinc finger [48]. This mutation reduces DNA binding affinity and diminishes transactivation of target gene expression by Gata1 [48]. The *gata*1^*T*301*K*/*T*301*K*^ fish had defective primitive erythropoiesis but normal definitive hematopoiesis. By combining the T301K allele with the Gata1 null allele of *vlad tepes*, we were able to generate an allelic series with different Gata1 activity levels, listed in the descending order: *gata*1^+/+^, *gata*1^+/*T*301*K*^, *gata*1^+/*vlt*^, *gata*1^*T*301*K*/*T*301*K*^, *gata*1^*T*301*K*/*vlt*^, *gata*1^*vlt*/*vlt*^. Analysis of fish with these genotypes demonstrated that erythropoiesis during primitive hematopoiesis requires higher activity level of Gata1 than erythropoiesis and thrombopoiesis during definitive hematopoiesis [48].

## 5. Concluding Remarks

**Figure 1:**
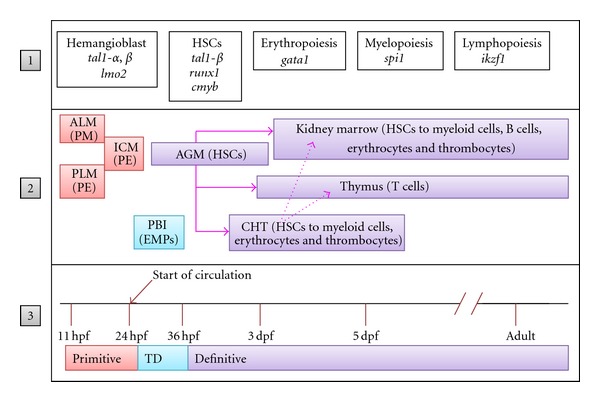
A schematic of overall view of zebrafish hematopoiesis with shifting sites, types of cells produced at each site, and genes involved, shown in 3 tiers as described below. Tier 1: lineage-specific transcription factors that control primitive and definitive hematopoiesis in zebrafish. Tier 2: the sites of action during each stage of hematopoiesis and the types of cells produced at each of the sites. The site boxes are color matched with waves of hematopoiesis and temporally placed according to the developmental stages in Tier 3. Tier 3: the time scale depicting the stage of development in hpf (hours postfertilization) and dpf (days postfertilization) and different waves of hematopoiesis. The abbreviations used are as follows: ALM: anterior lateral mesoderm, PLM: posterior lateral mesoderm, PBI: posterior blood island, AGM: aorta-gonad-mesonephros, CHT: caudal hematopoietic tissue, PM: primitive macrophages, PE: primitive erythrocytes, HSCs: hematopoietic stem cells, TD: transient definitive wave.

Depicted in Figure 1 is a schematic of the overall view of zebrafish hematopoiesis emerging from these studies. It is clear from the above-mentioned studies that zebrafish has played a significant role in our understanding of the genetic controls of hematopoiesis, particularly the dosage-specific requirements during different stages. The viability to adulthood with multi-lineage hematopoiesis in *runx1* knockout zebrafish clearly demonstrated that Runx1 is dispensable for adult hematopoiesis. Similarly, Ikzf1 was found to be dispensable for adult lymphopoiesis. On the other hand, Cmyb was found to be essential for adult hematopoiesis, while dispensable for larval definitive stage. Genetic mutants need to be generated for *spi1* to elucidate its exact role in maintaining proper balance between adult erythropoiesis and myelopoiesis. 

Proper functioning of the genetic controls regulating hematopoiesis is crucial for normal development of all the blood lineages. Mutations in critical genes at many of the steps lead to leukemogenesis. Thus, adult viable mutant zebrafish would allow us to understand the process of leukemogenesis. Furthermore, the recent application of next generation sequencing technologies to a variety of leukemia samples have led to the identification of several new genes mutated in leukemias [159, 160]. We anticipate that understanding their roles in normal hematopoiesis using the many advantages of the zebrafish model for hematopoiesis research would aid in therapeutic advances in the coming years.

## Figures and Tables

**Table 1:** Lineage-specific mutant and transgenic lines for zebrafish hematopoiesis research.

Lineage	Marker	Mutant lines		Transgenic lines	
		Mutant designation and mutation type	References	Line designation	References
Hemangioblast	*tal1/scl*	t21384, K183X	[38]	PAC-tal1:GFP 5.0tal1:EGFP	[39, 40]
	*lmo2*	None		lmo2:EGFP lmo2:DsRed	[41]

EMPs	*runx1*	hg1, W84X	[42, 43]	runx1P1:EGFP	[44]

	*runx1*	hg1, W84X	[42, 43]	runx1P2:EGFP	[44]
HSCs	*cmyb*	t25217, I181N hkz3, truncation in transactivation domain	[45] [46]	cmyb:EGFP	Developed by the Zon lab, used in [47]
	*cd41*	None		cd41:GFP	[33, 34]

Erythropoiesis	*gata1*	m651 (*vlad tepes*), R339X hg2, T301K	[23] [48]	gata1:GFP gata1:DsRed	[37, 49]

Myelopoiesis: GMPs	*spi1*/*pu.1 *	None		spi1:EGFP zpu.1:EGFP	[50, 51]

	*mpx*	None		mpx:GFP	[52]
Myelopoiesis: Neutrophils, Macrophages, Monocytes	*lyz*	None		lyz:EGFP lyz:DsRed	[53]
	*mpeg1*	None		mpeg1:EGFP mpeg1:mCherry	[54]

	*rag1*	t26683, R797X	[55]	rag1:GFP	[56]
Lymphopoiesis	*lck*	None		lck:EGFP	[57]
	*ikzf1/ikaros*	t24980, Q360X	[58]	ikzf1:GFP	[59]
